# Supplementary material for: The Evolution of tRNA Copy Number and Repertoire in Cellular Life
Source: Genes (Basel). 2022 Dec 22;14(1):27. doi: 10.3390/genes14010027 (PMC9858662; doi:10.3390/genes14010027)
Supplement: Supplementary file 1 [file genes-14-00027-s001.zip › genes-2070981-supplementary.pdf]

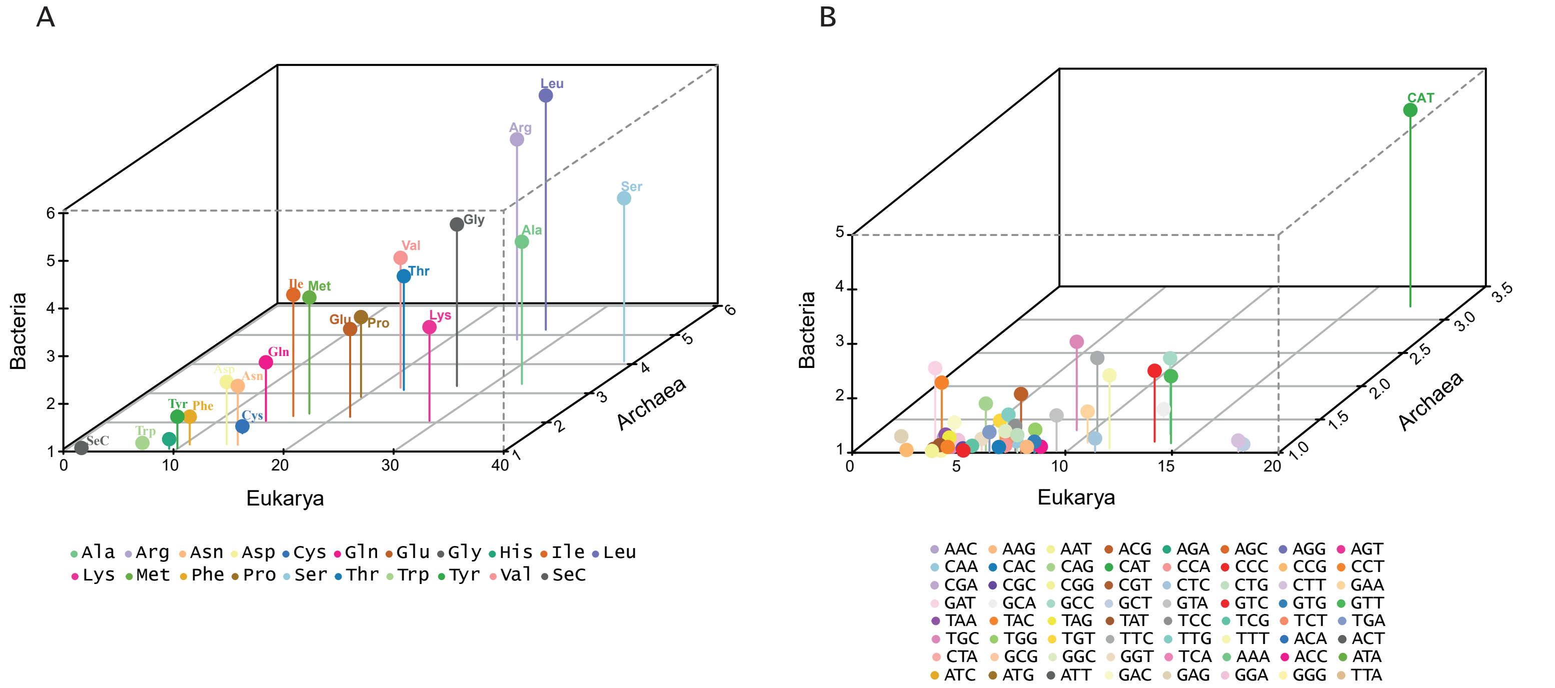

Supplemental Figure S1. Correlation of tRNA gene count among the three domains of life.
Mean tRNA gene count per isoacceptor (A) and per anticodon (B) per genome in Bacteria (z), Eukarya (x) and Archaea (y). The tRNA CN per isoacceptor is conserverd among the domains while the most abundant tRNA anticodon is MettRNA(CAT).

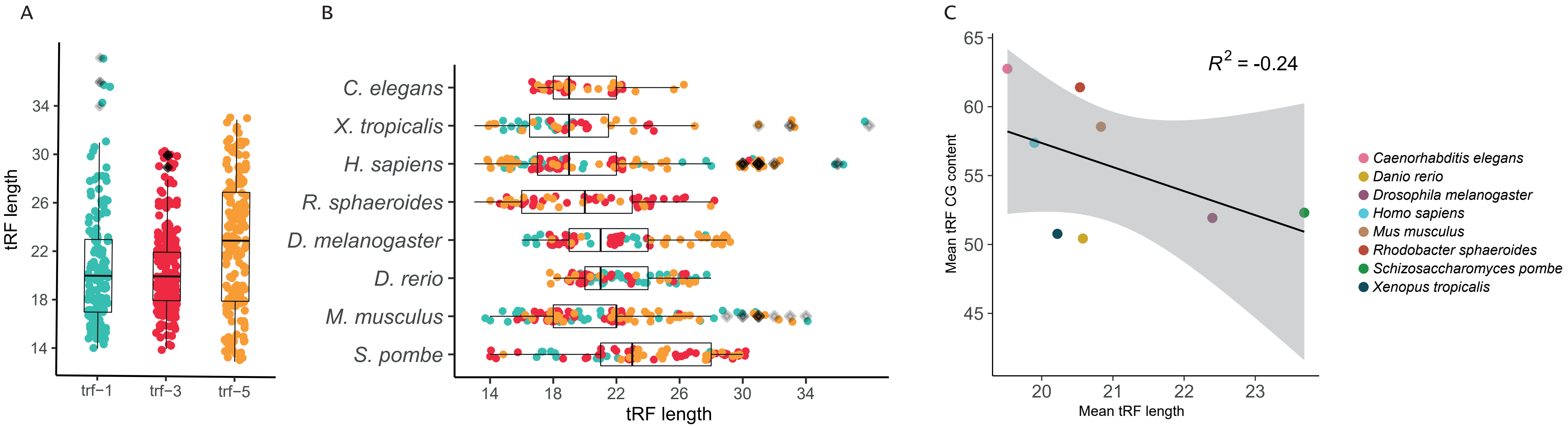

Supplemental Figure S2. Diversity of tRNA-derived RNA fragments (tRFs) length in selected genomes.
(A) Distribution of length per tRF type (nt). (B) Distribution of length of tRFs types per species (nt). (C) Negative correlation between mean tRF CG content (%) and mean tRF length (nt).
